# Basal Cell Carcinoma Arises from Interfollicular Layer of Epidermis

**DOI:** 10.1155/2018/3098940

**Published:** 2018-09-26

**Authors:** Sukmawati Tansil Tan, Mahmud Ghaznawie, Peter J. Heenan, Ricky Dosan

**Affiliations:** ^1^Department of Dermatovenereology, Faculty of Medicine, Tarumanagara University, Jakarta, Indonesia; ^2^Department of Pathology, Faculty of Medicine, Hasanuddin University, Makassar, Indonesia; ^3^Department of Pathology, Australian Clinical Laboratory, Nedlands, Australia

## Abstract

**Background:**

BCC is currently the most common type of skin cancer in humans. Although having a low-grade malignancy and metastatic potential, BCC is locally aggressive and destructive. Despite numerous studies, the origin of BCC, whether arising from the follicular or interfollicular layer, remains controversial.

**Objectives:**

This study aims to evaluate whether BCC arises from the follicular or interfollicular layer by using immunohistochemical staining.

**Methods:**

Twenty-three specimens of superficial and nodular BCC at its very early stage were examined. The samples were immunohistochemically stained using BerEP4 antibody. The stained specimens were then examined and scored by 2 independent observers.

**Results:**

BerEP4 was found to be strongly positive in all BCC lesions, including a very early lesions budding off the basal layer of the epidermis.

**Conclusion:**

This study confirmed that the origin site of BCC is basal layer of epidermis. This finding suggests that BCC arises from the interfollicular epidermis.

## 1. Introduction

BCC is one of the most frequently occurring cancers and the most common skin cancers in humans [1]. In Australia, the incidence was reported to be at 500-1560 tumors per 100.000 per year [2, 3]. An estimated 900.000 to 1 million are diagnosed in America, 550.000 men to 350.000 women, with around a 2:1 ratio. This prevalence is projected to increase twice in every 25 years, with the ratio becoming 3:2 [4]. Most BCCs are located on the upper parts of the body, with 75-80% located on the face making BCC although only locally destructive cosmetically damaging [5–7].

Histopathological diagnosis of BCC shows palisading of columnar cells, although this characteristical appearance has been debated for considerable time. The study on the origin site of BCCs has been performed by numerous researchers relying on various morphological and immunohistochemistry markers of hair follicles [8–11].

Based on the newest studies, it was thought that BCCs arise from the constitutive activation of the HH pathway through either Ptch loss of function or Smo gain of function. Different mouse models of BCC using Ptch1 deletion or oncogenic SmoM2 mutant expression induce the formation of tumors that resemble superficial human BCC. The skin epidermis contains distinct types of SCs that contribute to the homeostasis of discrete regions of epidermis. Interfollicular epidermis is maintained by stem cells targeted by K14-CreER and committed progenitors targeted by Inv-CreER in tail, ear, back, and ventral skin epidermis. Activation of oncogenic HH signalling through SmoM2 expression or Patched1 deletion in these different tissues using K14-CreER, which targets both stem cells and committed progenitors, induces BCC formation [12].

A wide variety of theories on their origin has been presented during the last century; however the histogenetic origin of BCC remains controversial and requires further exploration. Hence this study aims to evaluate whether BCC arises from the follicular or interfollicular layer through the use of immunohistochemical markers on histopathological specimens of BCC at the early stages of their development in order to find its site of origin.

## 2. Materials and Methods

### 2.1. Tissue Samples

Twenty-three specimens of BCC lesions were obtained from Healthscope Laboratories, Perth, Australia, between 2010 and 2011 and selected based on the inclusion and exclusion criteria. All of the lesions were early stage BCC from primary tumor in BCC patients. Twenty lesions were of multifocal superficial BCC and three were of nodular BCC.

### 2.2. Immunohistochemistry

The paraffin-embedded tissue blocks were cut into 4 *μ*m sections; one tissue section was then stained with Hematoxylin and Eosin to identify whether they are early stage BCCs. The other tissue section was used for immunohistochemistry staining. Immunohistochemistry staining was done using automatic immunohistochemical stainer Ventana Roche BenchMark XT.

BerEP4 Ventana Batch #1106708A (1:50 dilution) was used as the immunohistochemical marker. Antigen antibody reactions were visualized by Ultraview Brown Counterstain DAB Detection Kit (Ventana Medical Systems).

All immunohistochemical stains were then examined and scored by 2 independent dermatopathologists. Scoring was determined by two observers for examining the cytoplasmic staining intensity and percentage of stained cells. Staining intensity scored 3 for strongly stained, 2 for moderately stained, 1 for weakly stained, and 0 for unstained types. Stained cells percentages scored 4 for >80%, 3 for 50-79.9%, 2 for 20-49.9%, and 1 for<20%. Total score for both results were then added up to determine the expression of BerEP4 as 6-7 for strongly positive, 4-5 for moderately positive, 1-3 for weakly positive, or 0 for negative types. Interobserver consistency was then analysed using the Kappa Test. If the result was less than 0.4, both of the observers were asked to reconsider their results. If not, then the data will proceed for statistics.

## 3. Results

A total of 23 BCC patients participated in this study as shown in Table 1. Based on gender, participants in this study were 56.52% men (n=13) and 43.46% women (n=10). Mean age was 68.73 with standard deviation 14.36. In this study 26.09% BCC lesions were located on face (n=6); others were located on ankle (8.7%, n=2), back (8.7%, n=2), lower leg (8.7%, n=2), shoulder (8.7%, n=2), upper leg (4.35%, n=1), and chest (4.35%, n=1). Twenty cases were superficial type (86.96%) BCC and only three cases were nodular type BCC (13.04%).

BerEP4 is a reliable immunohistochemistry marker for BCC and does not stain normal skin. BerEP4 staining was done to confirm that the sections were of early stage BCCs and the exact location of these early lesions.

**Figure 1:**
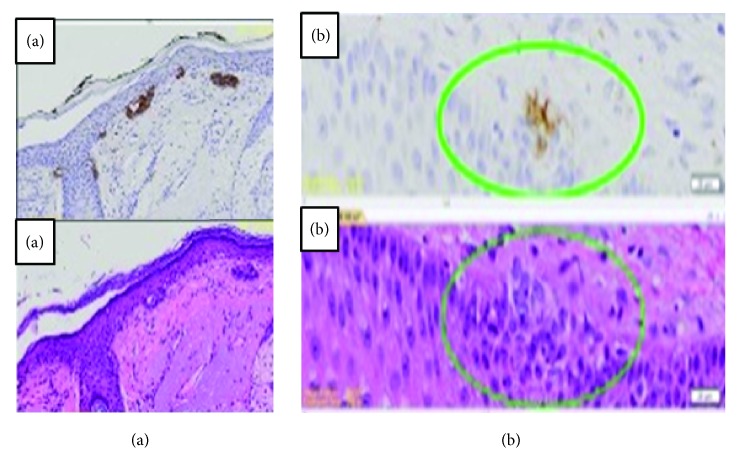
Hematoxylin-Eosin and BerEP4 immunohistochemistry staining in basal cell carcinoma. (a) Various BCCs with different size were seen around epidermal basal layer which grow towards dermis. They originate from infundibulum of hair follicle and basal epidermis. (b) Early lesions were visualized better in BerEP4 staining.

A strongly positive staining was found in all of BCC sections (23 of 23).  Figure 1 shows both Hematoxylin-Eosin and BerEP4 staining of BCC. In Figure 1(a) we found that the lesions are randomly spread in the interfollicular layer and intermittently along the basal layer of the epidermis.  Figure 1(b) shows that even very small microlesions containing only 4 to 15 BCC cells are visualized clearly and easy to find with BerEP4 staining. Lesions appear to bud from the basal epidermal layer and only a few are at the infundibulum of the hair follicles.

## 4. Discussion

The origin cell of BCC is still unclear and assumed to arise from basal cells, interfollicular cells [8, 9, 13, 14], or basal cells which differentiate to glandular cells [8, 14, 15]. Previous studies have hypothesized that BCCs arise from follicular germ cells such as the primary epithelial hair germ cell,* follicular germinative cells, *ORS of hair follicle, ORS of vellus hair follicles, hair matrix and primordial dermal adnexa, ORS of hair follicle of* embryonic hair germ*,* embryonic primary epithelial germ cell, *and pluripotent stem cell [9, 14, 16].

These conflicting results have made the origin cell of BCC remain uncertain. Most of the studies above have been done on mice's skin or cell culture in which BCC was induced through use of topical carcinogens or on late stage BCCs. The mouse epidermis has only 2 living cell layers with a relatively flat basal compartment, unlike the human epidermis which has many cell layers and an undulating basal compartment. Furthermore, mouse epidermis renewal is centered around highly ordered structures termed epidermal proliferative units, whereas in humans stem cells are dispersed along the basal and follicular compartments [17–19]. BCCs have also been proven to be induced by ultraviolet radiation, rarely through topical carcinogens only [20–23].

Previous study by Pinkus stated that all evidence for tumor origin based on connections of a sizeable growing tumor with one or other normal structures of the skin rests on a very shaky ground and tumor usually grows centrifugally from its site of origin. Almost any tumor that is visible to the naked eye is already too large and far advanced. To find information about the site of origin the smallest and earliest tumor should be used. Hence taking note of the limitations of Pinkus's studies and statement, this study used the presumably earliest lesions of BCC found on human skin [16]. In order to ensure that the earliest BCC specimens are used, the monoclonal antibody, BerEP4, immunohistochemistry staining was done. BerEP4 shows strong positive result at the cytoplasm and the membrane of the all BCC, anagen hair follicle, sebaceoma, eccrine glands, and the basosquamous carcinomas of the skin [10, 11, 24, 25].

Based on previous study in mice conditionally expressing constitutively active SmoM2 to activate Hedgehog signalling in different cellular compartments of the skin epidermis, Yousef et al. found that activation of SmoM2 in hair follicle bulge stem cells and their transient amplifying progenies did not induce cancer formation, showing that BCC does not originate from bulge stem cells, as previously thought. By using clonal analysis, Yousef et al. stated that BCC arises from long-term resident progenitor cells of the interfollicular epidermis and the upper infundibulum [1]. Two years later, by continuing previous study, it was discovered that human BCC also expresses genes of the Wnt signalling and embryonic hair follicle progenitors signatures. Wnt/*β*-catenin signalling was very rapidly activated following SmoM2 expression in adult epidermis and coincided with the expression of embryonic hair follicle progenitors markers. Deletion of *β*-catenin in adult SmoM2-expressing cells prevents embryonic hair follicle progenitors reprogramming and tumor initiation [26].

Another study in mice by Sanchez-Danes et al. shows that the proliferative hierarchical organization of skin epidermis is a key determinant of tumor development, with only interfollicular epidermis stem cells and not committed progenitors competent to initiate BCC following oncogenic HH signalling. Even though committed progenitors derived clones survive and proliferate for months, they were robust to BCC transformation and invasion and stayed in a protumorigenic state. It was suggested that the developmental stage of progenitors may also dictate competence for tumor initiation [12].

Interfollicular epidermis stem cells reside solely in the interscale region and have the regionalized competence to initiate large and invasive BCCs. Oncogene expression in stem cells leads to a more rapid clonal expansion as compared to committed progenitors for two main reasons: the maintenance of hierarchical organization in early preneoplastic lesions, leading to increased symmetric self-renewing divisions, and the combined resistance to apoptosis and enhanced proliferation of stem cell derived preneoplastic lesions, leading to a more effective growth rate. Both allowed stem cell targeted tumors to escape the dormant state that characterized committed progenitors targeted preneoplastic lesions and thereby progress to an invasive phenotype [12].

In this study, all 23 sections and 394 lesions of BCC show 100% strong positive staining by the BerEP4 antibody. Even for the earliest BCC lesion with only 4-15 cells BerEP4 clearly shows that BCC appears from basal cell in the basal layer of epidermis (interfollicular) and infundibulum of hair follicle with some groups of BCC cells budding down from the BCC basal layer, infiltrating into dermis and connected together inside the dermis. The three specimens of nodular BCC also showed that the BCC grows towards the dermis, while still having a part attached to the basal epidermis.

These findings indicate that histogenetically the origin cell of BCC is from the basal cell of epidermis, located at the interfollicular epidermis and infundibulum of the hair follicle distributed along the basal layer, supporting the theories that the origin of BCC arises from the basal cell layer of the interfollicular epidermis [8, 27], not the ORS and hair follicles [15, 28].

## 5. Conclusion

The results of this study showed that the origin site of BCC is to arise in the basal layer of interfollicular epidermis. BerEP4 can be used as a potential marker to detect very early lesion of BCC which is not clearly visible by standard histopathological staining. Because of that, BerEP4 can also be used to evaluate surgical margin after tumor surgery to determine whether it is clear from BCC. Further studies which identify stem cell origin of BCC are needed to answer many conflicting results from previous studies.

## Figures and Tables

**Table 1:** Patient characteristics.

Variable	n (%)
*Gender*	
Man	13 (56.52%)
Woman	10 (43.46%)

*Age*	68.73±14.36

*Location*	
Face	6 (26.09%)
Ankle	2 (8.7%)
Back	2 (8.7%)
Lower leg	2 (8.7%)
Neck	2 (8.7%)
Nose	2 (8.7%)
Scalp	2 (8.7%)
Shoulder	2 (8.7%)
Upper leg	1 (4.35%)
Chest	1 (4.35%)

*Type*	
Nodular	3 (13.04%)
Superficial	20 (86.96%)

## Data Availability

The histopathological data used to support the findings of this study are available from the corresponding author upon request.

## Abbreviations

BCC: Basal cell carcinoma
ORS: Outer root sheath
HH: Hedgehog
Ptch: Patched
Smo: Smoothened.
